# Analysis of Current Variation with Work Function Variation in L-Shaped Tunnel-Field Effect Transistor

**DOI:** 10.3390/mi11080780

**Published:** 2020-08-15

**Authors:** Jang Hyun Kim, Hyun Woo Kim, Young Suh Song, Sangwan Kim, Garam Kim

**Affiliations:** 1 School of Electrical Engineering, Pukyong National University, Busan 48513, Korea; janghyun@pknu.ac.kr; 2 Department of Electrical and Computer Engineering, Seoul National University, Seoul 08826, Korea; hyunoo1218@naver.com (H.W.K.); sys1413@snu.ac.kr (Y.S.S.); 3 Department of Computer Science, Korea Military Academy, Seoul 01805, Korea; 4 Department of Electrical and Computer Engineering, Ajou University, Suwon 16499, Korea; 5 Department of Electronic Engineering, Myongji University, Yongin 17058, Korea

**Keywords:** tunnel field-effect transistor (TFET), L-shaped TFET, high-κ/metal gate (HKMG), work-function variation (WFV), band-to-band tunneling, on-state current (*I*_ON_) variation, hump current (*I*_HUMP_) variation, ambipolar current (*I*_AMB_) variation

## Abstract

In this paper, an investigation is performed to analyze the L-shaped tunnel field-effect transistor (TFET) depending on a gate work function variation (WFV) with help of technology computer-aided design (TCAD) simulation. Depending on the gate voltage, the three variations occur in transfer curves. The first one is the on-state current (*I*_ON_) variation, the second one is the hump current (*I*_HUMP_) variation, and the last one is ambipolar current (*I*_AMB_) variation. According to the simulation results, the *I*_ON_ variation is sensitive depending on the size of the tunneling region and could be reduced by increasing the tunneling region. However, the *I*_HUMP_ and *I*_AMB_ variations are relatively irrelevant to the size of the tunneling region. In order to analyze the cause of this difference, we investigated the band-to-band tunneling (BTBT) rate according to WFV cases. The results show that when *I*_ON_ is formed in L-shaped TFET, the BTBT rate relies on the WFV in the whole region of the gate because the tunnel barrier is formed in the entire area where the source and the gate meet. On the other hand, when the *I*_HUMP_ and *I*_AMB_ are formed in L-shaped TFET, the BTBT rate relies on the WFV in the edge of the gate.

## 1. Introduction

Recently, an L-shaped tunnel field-effect transistor (TFET) has attracted the attention of a lot of researchers as a substitutional device for a metal-oxide-semiconductor (MOS) field-effect transistor (MOSFET) [1,2,3,4,5,6]. The L-shaped TFET features a mesa-shaped structure and an intrinsic Si region located between the source and gate dielectric layer to obtain high band-to-band tunneling (BTBT) due to the larger tunneling area than the planar TFET. The L-shaped TFET has remarkable advantages for low-voltage operation due to its small subthreshold swing (*S*) of less than 60 mV/dec, low-level OFF-state current (*I*_OFF_) and high complementary MOS (CMOS) compatibility [7,8,9,10]. Based on the characteristics, the electrical performance of the L-shaped TFET can be more improved dramatically by applying the high-κ/metal gate (HKMG) technology [11,12,13]. Thus, it is expected that the L-shaped TFET is applicable to the real industry. However, the application of HKMG brings a work-function (WF) variation (WFV) issue due to the non-uniformity of metal gate grains in orientation depending on the fabrication processes [14,15,16,17]. Therefore, in order to apply the TFET to the real application, the WFV in TFET should be investigated. Although there are several studies about the WFV effects on TFET, they have some common issues. The mentioned papers have focused on variation in electrical characteristics (e.g., threshold voltages (*V*_T_) and ON-state current (*I*_ON_)) in general structures (e.g., planar, fin, nanowire) and have not proposed the improvement of WFV in TFET [14,15,16,17,18].

This paper aims to study the effects of WFV in L-shaped TFET with the help of technology computer-aided design (TCAD) simulation. The L-shaped TFET is expected to improve the WFV due to the large tunneling area. Because the WFV has been studied, we know that the channel area and the WFV have a high correlation [19,20]. The contents of this paper are as follows. In Section II, the structure and dimension of the studied L-shaped TFET are explained. The WFV induced by the grain of the metal gate is set reflecting the actual gate physical properties. In Section III, the quantitative analysis is performed by confirming the location of metal grains and BTBT rate to monitor the variation of *I*_ON_, hump current (*I*_HUMP_) and ambipolar current (*I*_AMB_) of the L-shaped TFET.

## 2. Device Structure

**Figure 1:**
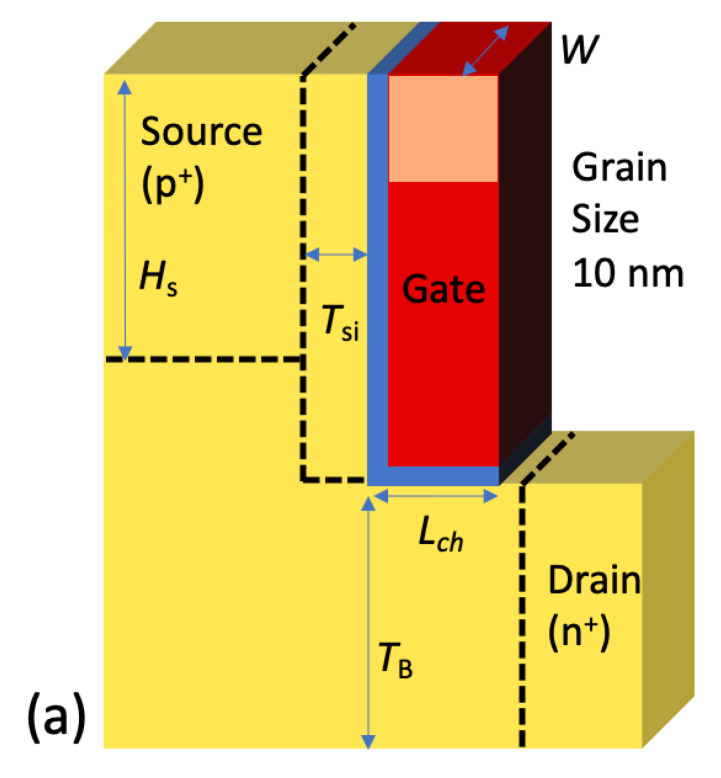

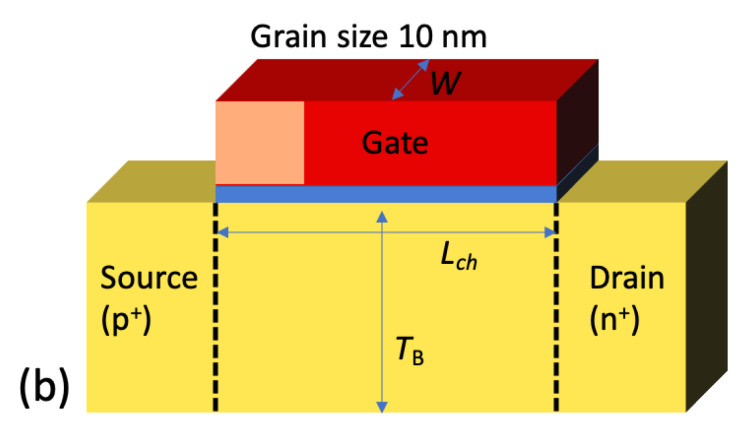
Schematic diagram of (**a**) the L-shaped tunnel field-effect transistor (TFET) and (**b**) the planar TFET. The L-shaped TFET features a vertical- band-to-band tunneling (BTBT) (parallel to the gate-field direction) in the intrinsic Si layer.

The structure of L-shaped TFET for WFV analysis is shown in Figure 1a. It features that thin intrinsic Si is deposited to restrict tunnel width for enhancing BTBT. All of the source, drain and channel materials consist of Si. The body thickness (*T*_B_) of 20 nm, the lateral channel length (*L*_ch_) of 50 nm, vertical tunneling thickness (*L*_tun_) of 6 nm and the SiO_2_ gate oxide thickness (*T*_OX_) of 1 nm are applied. p-type body doping (*N*_B_) of 1 × 10^17^ cm^−3^ is set. Then, both Si source and drain doping concentrations (*N*_S_, *N*_D_) are set as 1 × 10^20^ cm^−3^ with opposite doping types Boron and Arsenic. For confirming the effect for the area of the tunneling barrier, the source height (*H*_S_) is varied from 10 nm to 50 nm. The gate area is split into 10 nm × 10 nm units considering the grain size of TiN and it is assumed to be an identical square shape [21]. In the real fabrication process, the sputtered TiN is mainly crystallized in <200> (60%) and in <111> (40%) which correspond to 4.6-eV and 4.4-eV WFs, respectively [19,22]. In order to compare with planar TFET as a control group, the planar TFET has the same parameter for *W*, *T*_B_, *L*_ch_, *N*_S_, *N*_D_ and *N*_B_ (Figure 1b). All the specifications are summarized in Table 1.

The characteristics of the L-shaped TFET is simulated by the Synopsys Sentaurus^TM^. The Shockley–Read–Hall (SRH) and dynamic nonlocal BTBT model are used for accurate characteristics [23,24]. The dynamic nonlocal BTBT model is essential to examine lateral- and vertical- BTBT in the L-shaped TFET, since it can dynamically determine and calculate all tunneling paths based on the energy band profile [3,25,26,27]. In detail, the BTBT model calibrated with experimental results [28]. The BTBT generation rate per unit volume (G) defined as

(1) $$G=A{\left(\frac{F}{F_{0}}\right)}^{P}\exp\left(-\frac{B}{F}\right)\;\;\;$$

in the uniform electric field limit where *F*_0_ = 1 V/m and *P* = 2.5 for indirect tunneling [29]. The prefactor (*A*) and the exponential factor (*B*) are Kane parameters while the *F* is electric field [30,31]. The extracted *A* and *B* parameters of the BTBT model in Si TFET are 4×10^14^ cm^−1^s^−1^ and 9.9×10^6^ V/cm, respectively. Additionally, modified local density approximation (MLDA) is used for including quantum phenomena [32,33]. The MLDA model is needed to calculate the confined carrier distributions, especially inside the ultra-thin intrinsic Si tunnel region in which BTBT occurs. All the models are summarized in Table 2.

## 3. Results and Discussion

**Figure 2:**
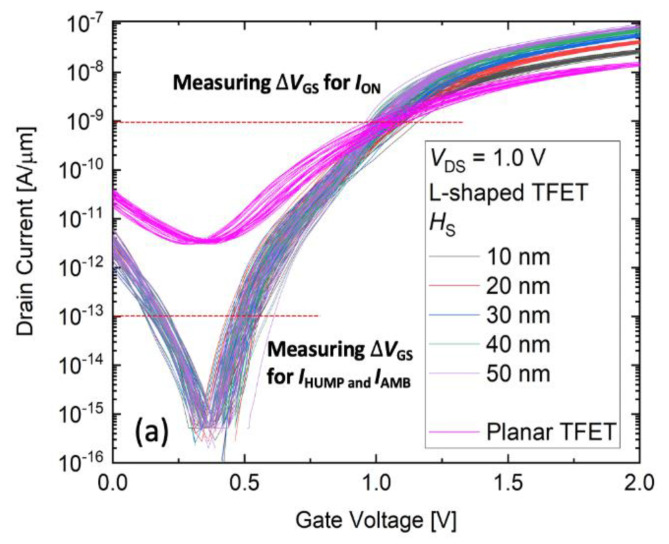

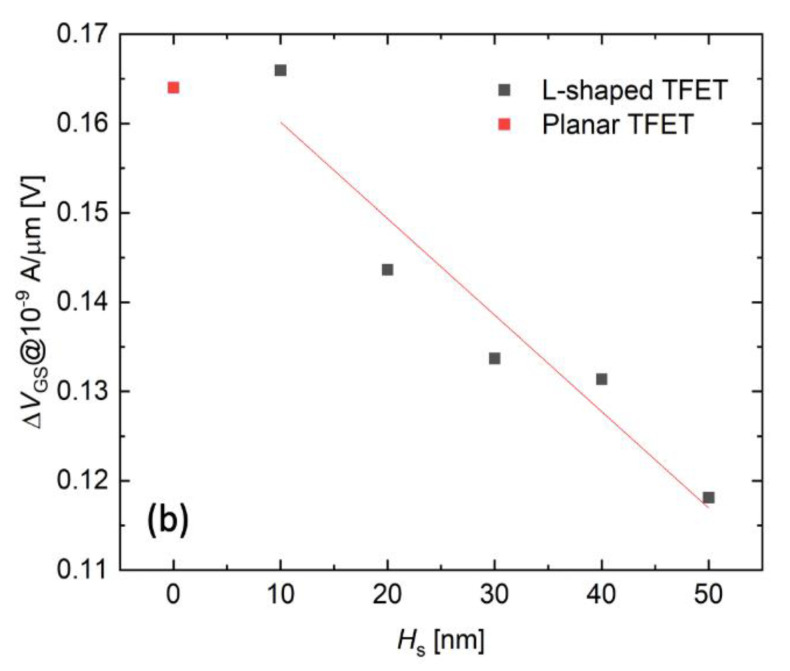
(**a**) Transfer curves of the L-shaped TFET. In each case of *H*_S_, 30 random samples for WFV are generated. (**b**) Dependency on *H*_S_ and *I*_ON_ variation.

**Figure 3:**
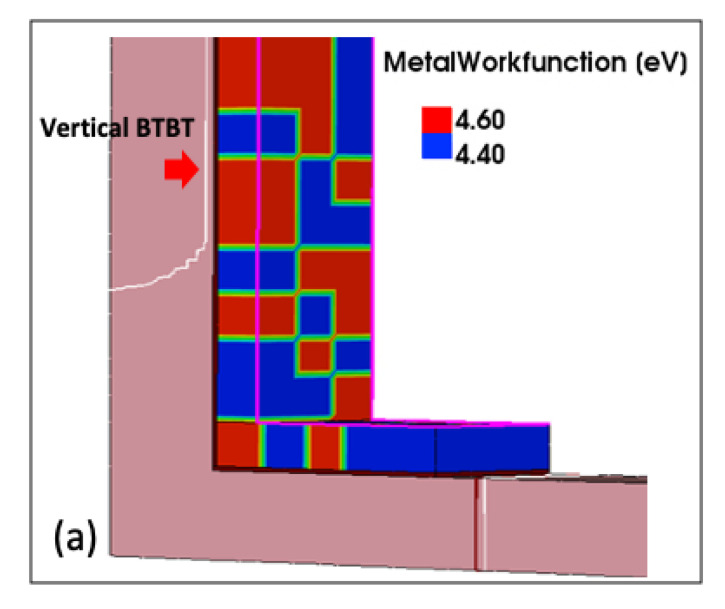

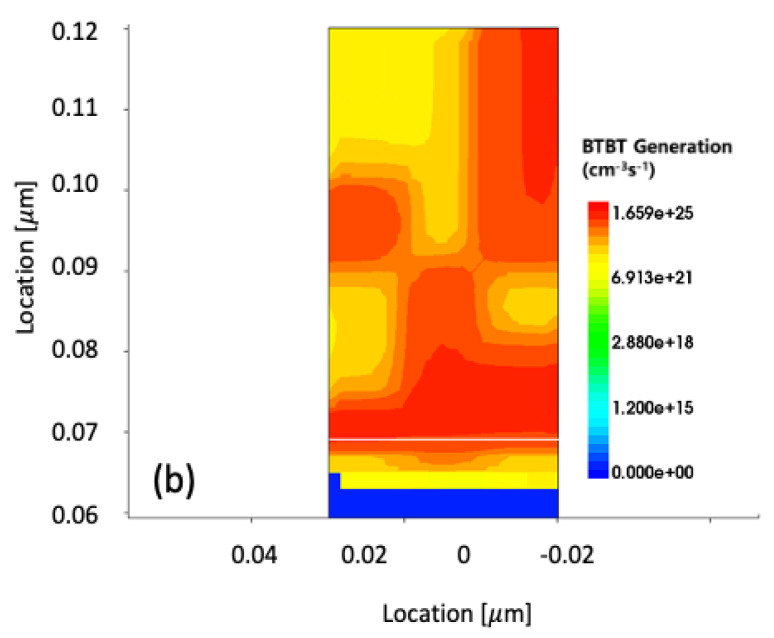
(**a**) TiN-grains distribution in gate area (**b**) BTBT rate on source region. The vertical-BTBT generated in the source area can be found to be high.

Figure 2a shows the transfer characteristics of the planar TFET and L-shaped TFET with various source heights (*H*_S_) at 1.0 V of drain voltages (*V*_DS_). In each case of *H*_S_, the 30 samples are simulated, and each sample contains randomly generated TiN grains in the gate. The *I*_ON_ of the L-shaped TFET increases as the *H*_S_ increases. The result shows that the L-shaped TFET can improve the weak drivability of *I*_ON_, which is a weakness of TFET. In addition, the average *S* (*S*_avg_), defined as the average inverse slope of the transfer curve while *I*_D_ changes from 10^−15^ μA/μm to 10^−11^ μA/μm, is shown from 35 to 45 mV/decade for the L-shaped TFET which means that the L-shaped TFET suggests possible applications for the low power operation [34,35]. In transfer curves, we measure three regions: *I*_ON_, *I*_HUMP_ and *I*_AMB_ variations. Each variation extracted the difference between the maximum and minimum values of the *V*_GS_ values represented by each sample when the *I*_ON_, *I*_HUMP_ and *I*_AMB_ are formed. Firstly, the *I*_ON_ variation is investigated. The variation of *I*_ON_ is extracted from Figure 2a at 10^−9^ A/μm of drain current (*I*_D_). For the *I*_ON_ variation, it is found that the greater the *H*_S_ value, the smaller the *I*_ON_ variation and the *I*_ON_ variation of the L-shaped TFET could be reduced compared with that of the planar TFET (Figure 2b). For the planar TFET, the tunnel barrier that determines the current, is formed only in the area adjacent to the source and channel [14,36]. This means that the *I*_ON_ variation in planar TFET relies on the WFV in areas adjacent to the source, not on the whole area of the gate. However, for the L-shaped TFET, the tunneling area affected by the WFV is relatively wider than that of the planar TFET because the tunnel barrier is formed in the entire area where the source and gate meet [1]. Figure 3a shows a sample produced by a random WFV. Inside the gate, 4.4 eV and 4.6 eV grains are placed. Based on this sample, the vertical-BTBT generated in the source area can be found to be high where the grain of 4.4 eV is located (Figure 3b). In other words, when the tunnel barrier has a large area, the BTBT rate can have an average effect.

**Figure 4:**
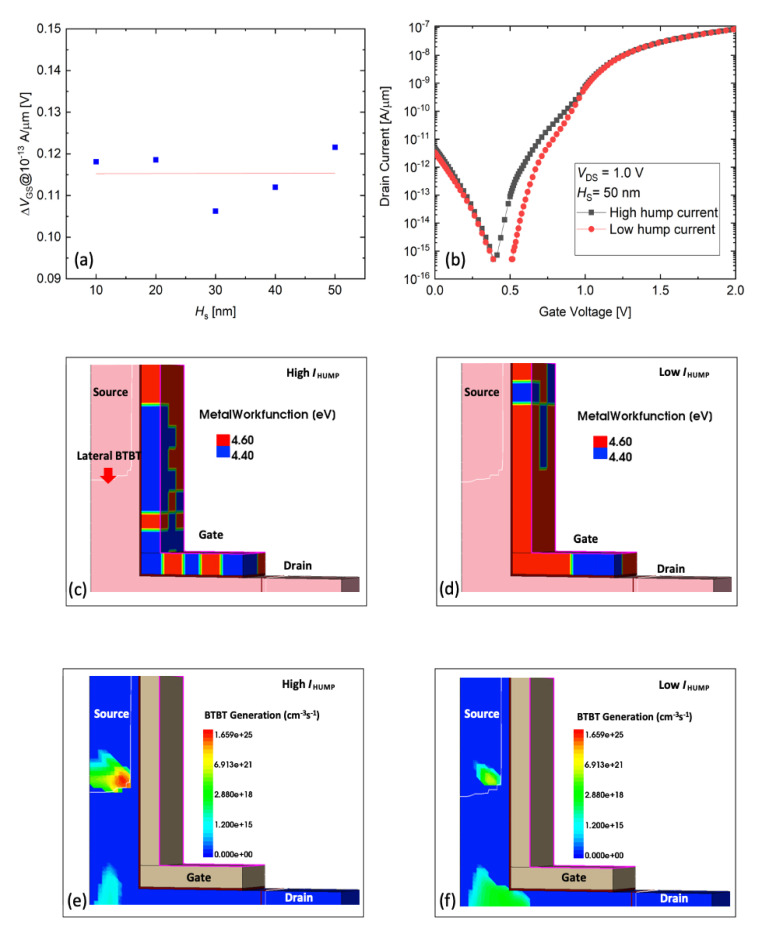
(**a**) *I*_HUMP_ variation dependency on *H*_S_. (**b**) Transfer characteristics with high and low *I*_HUMP_. TiN-grains distribution in gate area for (**c**) high *I*_HUMP_ sample and (**d**) low *I*_HUMP_ sample. BTBT rate for (**e**) high *I*_HUMP_ sample and (**f**) low *I*_HUMP_ sample.

Next, the *I*_HUMP_ variation is investigated. For *I*_HUMP_, as reported in the previous papers, the L-shaped TFET has vertical-BTBT and lateral-BTBT [37,38,39]. The lateral-BTBT is formed at low gate bias (*V*_GS_) due to low *V*_T_, resulting in a hump phenomenon. As shown in Figure 4a, the variation of *I*_HUMP_ is extracted from Figure 2a at 10^−13^ A/μm of *I*_D_ and it shows similar variations regardless of the change in the *H*_S_ value. To confirm this, we investigate two cases where the hump effect is high and low (Figure 4b). In the transfer curves, the two samples have almost the same *I*_ON_ values, while the samples have different *I*_HUMP_ values. For the sample with a high hump effect shown in Figure 4c, a high BTBT occurs mainly at the edge of the source area (Figure 4e). On the contrary, for the sample with a low hump effect shown in Figure 4d, a low BTBT is confirmed (Figure 4f). Specifically, the lateral-BTBT is measured highly where 4.4 eV grain is located in the source edge region, which causes a hump effect. As a result, the hump phenomenon is not related to the intrinsic Si area because tunneling occurs only at the edge of the source region.

**Figure 5:**
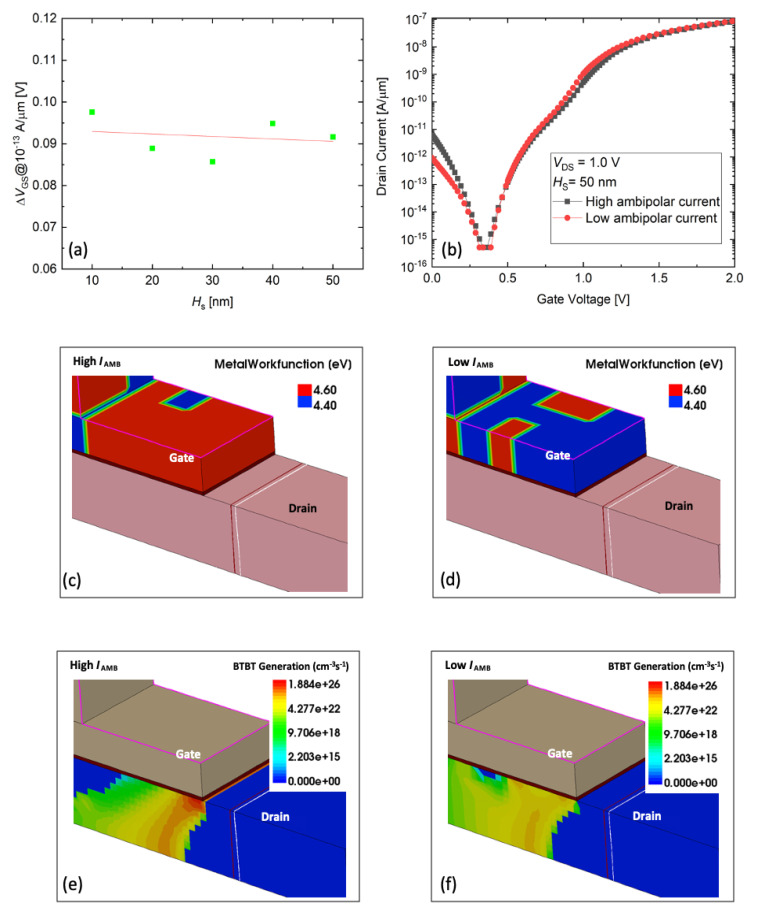
(**a**) *I*_AMB_ variation dependency on *H*_S_. (**b**) Transfer curves with high and low *I*_AMB_. TiN-grains distribution in gate area for (**c**) high *I*_AMB_ sample and (**d**) low *I*_HUMP_ sample. BTBT rate for (**e**) high *I*_HUMP_ sample and (**f**) low *I*_AMB_ sample.

Finally, the variation of the *I*_AMB_ is investigated. As shown in Figure 5a, the variation of *I*_AMB_ is extracted from Figure 2a at 10^−13^ A/μm of *I*_D_. For *I*_AMB_ variation, little dependency on the L-shaped TFET is shown with *H*_S_ values. To confirm this, we investigate two cases where the hump effect is high and low (Figure 5b). In the transfer curves, it shows almost the same current in all regions except the *I*_AMB_. As a result of confirming the high and low *I*_AMB_ samples (Figure 5c,d), it can be found that the BTBT occurs mainly in the edge of the drain at the gate, where there is a 4.6 eV grain (Figure 5e, Figure 5f).

In conclusion, the L-shaped TFET could be a solution to reduce *I*_ON_ variation for WFV in TFET. However, the WFV reduction effect is not seen on whole electrical performance, especially for the *I*_HUMP_ and *I*_AMB_. These parameters are only affected by the edge region of the gate. Thus, for the real application of the L-shaped TFET, the WFV improvement should proceed through simultaneous applications of gate underlap technology that can reduce *I*_AMB_ and the dual WF gate, reducing the *I*_HUMP_ [25,40,41].

## 4. Conclusions

In this paper, the L-shaped TFET is investigated for WFV. We investigate all the variations divided into *I*_ON_, *I*_HUMP_, *I*_AMB_ and study each variation. The improved results are shown for WFV in the L-shaped TFET versus the planar TFET because the L-shaped TFET uses the wide tunnel barrier region. Based on these results, it was confirmed that increasing the tunnel area in the TFET device can be a method to decrease WFV. Therefore, the *I*_ON_ variation could be reduced by an increase in the tunneling region. However, the *I*_HUMP_ and *I*_AMB_ variations are relatively irrelevant to the size of the tunneling region.

## Figures and Tables

**Table 1:** Device parameters of devices used for technology computer-aided design (TCAD) simulation.

Parameters	Value	
Device	L-shaped TFET	Planar TFET
Source doping concentration (*N*_S_)	10^20^ cm^−3^ (p-type)	
Drain doping concentration (*N*_D_)	10^20^ cm^−3^ (n-type)	
Body doping concentration (*N*_B_)	10^17^ cm^−3^ (p-type)	
Channel length (*L*_ch_)	50 nm	
Channel width (*W*)	30 nm	
Metal grain size	10 nm	
Intrinsic layer thickness (*T*_Si_)	6 nm	none
Gate oxide thickness (*T*_OX_)	1 nm	
Drain voltage (*V*_D_)	1.0 V	
Source height (*H*_S_)	varied	

**Table 2:** Models in TCAD simulation.

Definition	Model
Bandgap narrowing	Old slot boom
Fermi Statistic	Fermi
Phonon scattering	Constant mobility
Multi-valley for quantum confinement	MLDA
SRH recombination	SRH/TAT
Nonlocal BTBT	Band to Band
WFV	4.6/4.4 eV (60%/40%) (Random generation)
